# Microsurgery *vs.* radiosurgery for the treatment of multiple metastases in the brain: a retrospective cohort study

**DOI:** 10.20892/j.issn.2095-3941.2020.0598

**Published:** 2021-09-08

**Authors:** Qun Liu, Qiang Yin, Yang Dong, Fengtong Li, Wenliang Li, Xiaoguang Wang

**Affiliations:** 1 Department of Neurosurgery and Neuro-Oncology, Tianjin Medical University Cancer Institute and Hospital, National Clinical Research Center for Cancer, Key Laboratory of Cancer Prevention and Therapy, Tianjin, Tianjin’s Clinical Research Center for Cancer, Tianjin 300060, China; 2 CyberKnife Center, Tianjin Medical University Cancer Institute and Hospital, National Clinical Research Center for Cancer, Key Laboratory of Cancer Prevention and Therapy, Tianjin, Tianjin’s Clinical Research Center for Cancer, Tianjin 300060, China

**Keywords:** Surgery, radiosurgery, brain metastasis, therapy, overall survival

## Abstract

**Objective:**

Multiple brain metastases are a severe condition for cancer patients. To date, no general consensus exists regarding the optimal treatment procedure for multiple brain metastases. Radiotherapy is the most commonly used treatment option. The role of surgical resection for multiple brain metastases is unclear. The aim of this study was to compare the outcomes of patients with multiple brain metastases treated with either surgery or stereotactic radiosurgery (SRS).

**Methods:**

The medical records of 279 consecutive adult patients with multiple brain metastases treated with either surgery (26 patients) or SRS (253 patients) were retrospectively reviewed. Propensity score matching was conducted to correct for discrepancies in the baseline characteristics, and 78 patients (26 receiving surgery and 52 receiving SRS) were chosen for comparison of outcomes, such as overall survival, local tumor control rate, and symptom improvement.

**Results:**

The tumor size in the surgery group was significantly greater than that in the SRS group after propensity score matching. However, the neurological recovery rate, incidence of leptomeningeal metastasis after surgery, 1-year local tumor control rate, and overall survival were not significantly different between groups.

**Conclusions:**

Our data demonstrate that surgery and radiosurgery have identical overall survival and local tumor control rates in patients with 2 to 4 brain metastases. Although SRS remains the primary and standard option for patients with brain metastasis, surgery offers several distinct advantages, such as establishing a diagnosis or relieving mass effects, and may additionally be beneficial in carefully selected patients with 2–4 brain metastases.

## Introduction

Brain metastases are the most commonly occurring tumors in the brain, and as many as 40% of adult patients with cancer will develop this life-threatening neurological problem at some stage^1,2^. The incidence rate ranges from 8.3 to 14.3 per 100,000, and is increasing because of the prolonged survival of cancer patients and improved screening^3^.

More radical approaches, such as surgery or stereotactic radiosurgery (SRS), are generally considered to improve the outcomes of patients with a single brain metastasis^4^. Many studies have shown no significant differences between surgery and stereotactic radiotherapy in this scenario^5^. The selection of treatment depends on many factors, including the location, size, and type of lesion^6^. However, more than 50% of brain metastases are multiple lesions at the time of first diagnosis^7^.

At present, no general consensus exists in treatment guidelines for multiple brain metastases^8^. Although whole-brain radiotherapy (WBRT) is the most common approach for patients with multiple brain metastases, stereotactic radiotherapy is increasingly being applied, because many studies have shown that stereotactic radiotherapy improves outcomes in patients with multiple brain metastases^9^. However, SRS may not be an option for all patients with multiple brain metastases, particularly those with large lesions that might be suitable for surgery. Resection of multiple nonadjacent lesions is considered to cause excessive morbidity and mortality and is generally avoided for patients with minimal life expectancy^6,10^. Only a few retrospective reports describing surgical treatment of multiple brain metastases have demonstrated a favorable median survival time and similar outcomes between single craniotomy and multiple craniotomy in patients with brain metastasis^11^. To date, no report has compared the outcomes between multiple craniotomies and SRS.

## Materials and methods

### Patients and treatments

We evaluated 279 consecutive adult patients with multiple brain metastases treated with either surgery (26 patients) or SRS (253 patients) at Tianjin Medical University Cancer Institute and Hospital between 2014 and 2018. The study was approved by the Ethics Committee of Tianjin Medical University Cancer Institute and Hospital. The committee also waived the need for informed consent from each patient. All clinical data were collected anonymously to maintain patient confidentiality.

The diagnosis of cerebral metastases was confirmed by magnetic resonance imaging (MRI).

The treatment decision of either surgery or SRS was based on clinical characteristics, such as tumor size, tumor location, and systemic disease status. The criteria for surgery were oligometastatic brain disease (2 to 4 lesions), Karnofsky performance status (KPS) ≥ 60, controlled primary disease, deteriorated clinical condition from cerebral disease, and substantial improvement after steroid administration. The postoperative treatment was decided by the surgeon according to the histologic sensitivity to therapy and the risk of local recurrence, such as the residual or dissemination signs on postoperative enhanced MRI, piecemeal resection, and insufficient surgery margin.

All patients in the SRS group were treated with a CyberKnife (Accuray Inc., Sunnyvale, CA, USA). The CyberKnife has a computer-controlled robotic arm with a 6 MV linear accelerator^12^. The treatment plan was established on the basis of the fusion of 1.5 mm slice thickness contrasted computed tomography and MRI images. The planning target volume was generated by extending 1.6 mm from the gross target volume, which was defined as the enhanced lesion on MRI. The number of treatments varied from 1 to 5 daily sessions, depending on the tumor size, location, and shape. Each treatment lasted from 30 to 90 min. All patients were asked to lie on the procedure table in supine position without anesthesia or contact while the robotic arm moved to treat all areas of the tumor.

### Outcomes

All patients were evaluated on the basis of the following parameters: sex, age, primary tumor, number of brain metastases, status of extracranial metastases, pretreatment KPS, neurological symptoms, and prior procedures. Preoperative and postoperative MRIs of each patient were obtained and reviewed. The tumor was approximated as an ellipsoid, and the volume was calculated according to measured values of x, y, and z on a contrast-enhanced MRI (V = xyz/2).

To overcome bias in the treatment choice and to adjust for differences in clinical characteristics, we conducted a case-matched study by using propensity score matching (2:1) for clinical factors (i.e., age, sex, pathology, primary tumor, interval between initial pathology diagnosis and treatment, KPS score, extracerebral metastases, neurological symptoms, and prior WBRT). A 0.1 caliper was used to match patients to the nearest neighbor. After propensity score matching, the baseline covariates did not significantly differ between groups.

The endpoints were overall survival (OS) and local progression of the treated tumor. Local tumor progression was defined as a 20% increase in the maximum diameter after treatment, as compared with the smallest maximum diameter of the enhanced lesion just before the treatment or after the treatment. For tumors 1 cm or less in maximum diameter, we did not record tumor progression even when a 20% increase in diameter was observed, as defined by Response Evaluation Criteria In Solid Tumors (RECIST)^13^. Local tumor progression categories included tumor recurrence, radiation necrosis, and mixed/undetermined.

### Statistical analysis

For the baseline characteristics, summary statistics are shown as frequencies and proportions for categorical data and as median and standard deviations for continuous variables. For categorical outcomes, Chi-squared test or Fisher’s exact test were used and 2-sided t-test were used for continuous variables. OS was analyzed with the standard Kaplan-Meier method. Univariable and multivariable analysis for the prognostic baseline clinical variables was performed with a Cox proportional hazard model.

A 2-tailed *P*-value less than 0.05 was considered statistically significant. All statistical analyses were performed in the R statistical program, version 3.4.0.

## Results

We identified 26 patients who received surgery for multiple brain metastases and 253 patients who received SRS for multiple brain metastases. The differences in clinical characteristics in the surgery group and SRS group before propensity score matching are summarized in **Table 1**. Propensity score analysis was applied in 78 patients (26 patients who received surgery and 52 patients who received SRS). After matching, the patient characteristics, including age, sex, KPS, symptoms, previous treatment, and other factors, were similar between the surgery group and SRS group (**Table 2**).

**Table 1:** Clinical characteristics of patients with multiple brain metastases treated with surgery or SRS before propensity score matching

Characteristics	Cases	Treatment		*χ* ^2^	*P*
		CK, *n*	Surgery, *n*		
Age, years				2.063	0.103
≤ 65	209	186	23		
> 65	70	67	3^a^		
Gender				0.854	0.355
Female	121	107	14		
Male	158	146	12		
Primary cancer				1.839	0.607
Non-small cell lung cancer	172	158	14		
Small cell lung cancer	25	21	4^a^		
Breast cancer	45	40	5		
Other	37	34	3^a^		
KPS				3.598	0.034*
< 70	40	40	0^a^		
≥ 70	239	213	26		
Metastasis outside CNS				6.485	0.011*
None	84	70	14		
Yes	195	183	12		
No. of metastases				2.476	0.386
2	199	177	22		
3	60	57	3^a^		
4	20	19	1^a^		
Prior WBRT				3.214	0.073
Yes	127	120	7		
None	152	133	19		
RPA class				9.013	0.011*
1	56	46	10		
2	183	167	16		
3	40	40	0^a^		
Neurological function				11.888	0.036*
Headache	96	85	11		
Motor weakness	61	53	8		
Speech	12	10	2^a^		
Vision loss	17	12	5		
Other	14	12	2^a^		
Normal	99	95	4^a^		
Site				15.595	0.016*
Frontal lobe	142	122	20		
Parietal lobe	121	111	10		
Temporal lobe	103	96	7		
Occipital lobe	95	84	11		
Cerebellum	104	95	9		
Thalamus	80	80	0^a^		
Brainstem	13	13	0^a^		
Time to BM, months				1.215	0.271
≤ 12	127	112	15		
> 12	152	141	11		

^a^Fisher’s test was used when *n* < 5; *P < 0.05.

**Table 2:** Clinical characteristics in propensity-score matched cohorts of patients with multiple brain metastases treated with surgery or SRS

Characteristics	Cases	Treatment		*χ* ^2^	*P*
		CK, *n*	Surgery, *n*		
Age, years				0.111	0.741
≤ 65	66	43	23		
> 65	12	9	3^a^		
Gender				0	1
Female	42	28	14		
Male	36	24	12		
Primary cancer				0.578	0.901
Non-small cell lung cancer	45	31	14		
Small cell lung cancer	13	9	4^a^		
Breast cancer	13	8	5		
Other	7	4^a^	3^a^		
KPS				0.39	0.547
< 70	3	3^a^	0^a^		
≥ 70	75	49	26		
Metastasis outside CNS				0.591	0.808
None	45	31	14		
Yes	33	21	12		
No. of metastases				0.938	0.626
2	63	41	22		
3	13	10	3^a^		
4	2	1^a^	1^a^		
Prior WBRT				0	1
Yes	22	15	7		
None	56	37	19		
RPA class				1.853	0.396
1	32	22	10		
2	43	27	16		
3	3	3^a^	0^a^		
Neurological function				1.833	0.175
Normal	21	17	4^a^		
Abnormal	57	35	22		
Site				10.956	0.089
Frontal lobe	48	28	20		
Parietal lobe	27	17	10		
Temporal lobe	23	16	7		
Occipital lobe	30	19	11		
Cerebellum	27	18	9		
Thalamus	12	12	0^a^		
Brainstem	6	6	0^a^		
Time to BM, months				0.058	0.809
≤ 12	42	27	15		
> 12	36	25	11		

^a^Fisher’s test was used when *n* < 5.

In the matching cohort, the size of the treated lesions in the surgery group (13,072 mm^3^, 485–65,120 mm^3^) was significantly greater than that in the SRS group (1,623 mm^3^, 42–58,036 mm^3^). In 26 patients, 57 lesions were resected, 84.6% (22 patients) underwent resection of 2 lesions, 11.5% (3 patients) underwent resection of 3 lesions, and 3.8% (1 patient) underwent resection of 4 lesions. In the surgery group, the primary tumor was lung cancer in 18 patients, breast cancer in 5 patients, malignant melanoma in 1 patient, colorectal carcinoma in 1 patient and renal carcinoma in 1 patient.

In the SRS group, patients who had not previously received WBRT were administered 18–22 Gy in 1 fraction, 26–30 Gy in 2 fractions, and 30–36 Gy in 3 fractions. Patients who had previously received WBRT were administered doses of 13–16 Gy in 1 fraction, 16–22 Gy in 2 fractions, and 24– 30 Gy in 3 fractions.

One patient in the surgery group developed intracranial infection after surgery. Two patients in the SRS group had transient radiogenic complications 1 week after SRS but fully recovered after steroid treatment. One patient in the SRS group had seizures.

Evaluation of neurological status 2 months after surgery or SRS indicated that 10 of the 11 patients with headaches fully recovered after surgery. Of 8 patients with motor weakness, 6 fully recovered, 1 partially recovered, and 1 remained the same before and after surgery. Of the patients who lost vision, 3 patients recovered, and 2 remained the same. In the SRS group, 13 of the 19 patients with headaches fully recovered, and 6 patients improved. Seven of the 14 patients with motor weakness fully recovered, 4 improved, and 3 remained the same. Overall, in the surgery group, 16 (77.2%) of the 22 patients with neurological deficits fully recovered, and in the SRS group, 22 (62.9%) of the 35 patients with neurological deficits fully recovered (*P* = 0.61).

**Figure 1:**
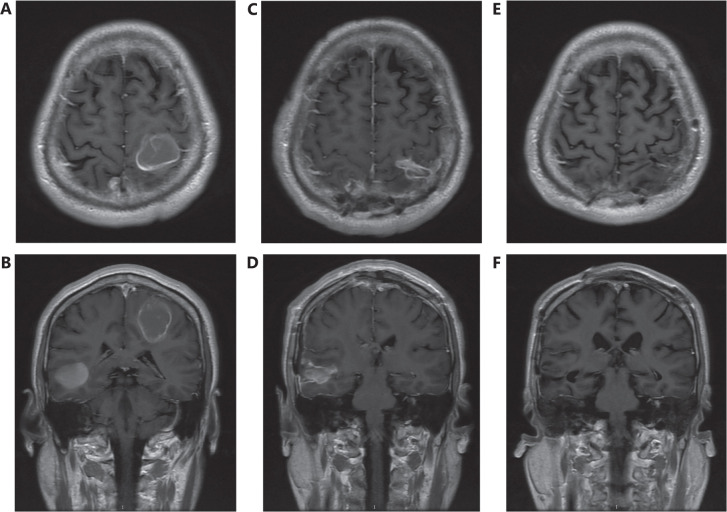
Preoperative axial (A) and coronal (B) T1 MRI with contrast, demonstrating 3 cerebral metastases: 1 in the left parietal lobe, 1 in the right parietal lobe, and 1 in the right temporal lobe. One month postoperative axial (C) and coronal (D) MRI showing total resection of all 3 metastatic lesions. One year postoperative axial (E) and coronal (F) MRI showing no tumor recurrence.

The multiple lesions from all 26 patients in the surgery group were completely resected, as demonstrated by MRI 1 or 2 weeks after surgery (**Figure 1**). In 19 patients who had not undergone WBRT before surgery, 7 received WBRT after surgery, 3 received surgery cavity SRS after surgery, 3 received targeted therapy effective in the brain, and 6 did not receive any effective treatment for brain metastasis after surgery. In 7 patients who had undergone WBRT before surgery, 3 received SRS after surgery. Four patients did not receive radiotherapy after surgery for brain metastasis.

The MRI scans showed local recurrence in 3 patients in the surgery group. The recurrent tumors were controlled in these patients after CyberKnife treatment (1 patient), repeated surgery (1 patient), or IMRT (1 patient). Eight patients in the SRS group exhibited local progression. Six of these patients received WBRT before or after SRS. Six of these patients were considered to have radiation necrosis, which was relieved after bevacizumab treatment. One patient received surgery for the recurrence, and 1 patient received repeat SRS. The 1-year local tumor control rate was not statistically significant different between the SRS group (84.6%) and the surgery group (88.5%).

**Figure 2:**
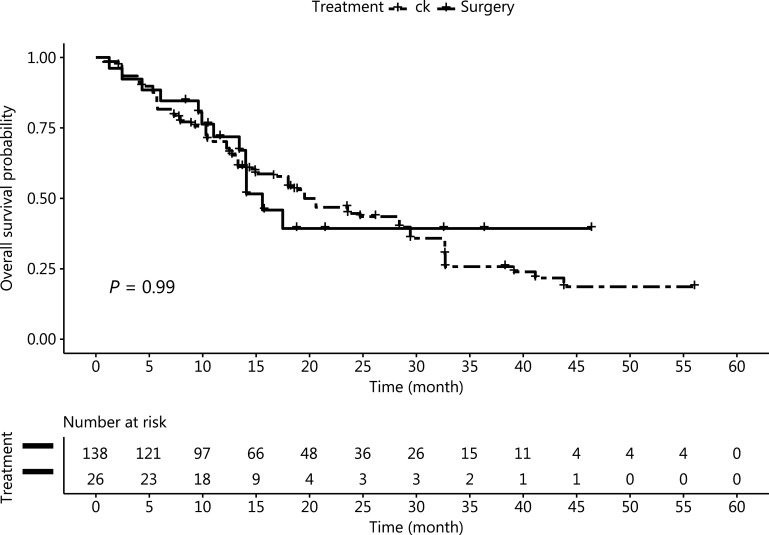
Overall survival after case matching, according to intention to treat; 26 patients received surgery, and 52 patients received SRS.

The incidence of leptomeningeal metastasis after treatment in the surgery group (19.2%, 5 cases) was slightly higher than that in the SRS group (13.5%, 7 cases). However, the difference was not statistically significant (*P* = 0.74). The median survival times in the surgery group and the SRS group were 17 months (1–46 months) and 16 months (1–56 months), respectively. The OS ratios after treatment at 1, 2, and 3-year were 55.8%, 23.1%, and 9.6%, respectively, in the SRS group; and 61.5%, 19.2%, and 11.5%, respectively, in the surgery group; these results did not significantly differ (**Figure 2**). The incidence of neurological death after SRS (6 cases, 11.5%) or surgery (2 cases, 7.7%) did not significantly differ between groups. Only 1 patient in the SRS group died because of recurrence of the treated tumor. None of the patients in the surgery group died because of local progression of the treated lesions.

Univariate analysis suggested that age (*P* = 0.028), KPS score (*P* = 0.012), RPA class (*P* = 0.005), and time to metastasis (*P* = 0.008) significantly predicted OS. These variables were included in the multivariate analysis, thus indicating that high RPA class (*P* = 0.01) was associated with poor prognosis in patients with multiple brain metastases (**Table 3**).

**Table 3:** Prognostic factors for overall survival of all patients in propensity-score matched cohorts after treatment

Covariate	Comparison	Univariate analysis		Multivariate analysis	
		*χ* ^2^	*P* value	*χ* ^2^	*P* value
Age, years	< 65 *vs.* ≥ 65	2.425	0.028*	1.03	0.14
Sex	Male *vs.* female	1.828	0.545		
RPA	1 *vs.* 2	3.289	0.005**	2.219	0.01*
KPS	< 80 vs. > 70	0.449	0.012*	0.511	0.457
No of lesions	2 *vs.* > 2	0.479	0.122		
Primary cancer	Lung *vs.* other	1.14	0.71		
	Lung *vs.* breast	0.695	0.417		
Prior WBRT	Yes *vs.* no	1.227	0.53		
Neurologic symptoms	Yes *vs.* no	1.543	0.27		
Extracranial metastasis	Yes *vs.* no	1.442	0.253		
Time to BM, months	< 12 months *vs.* ≥ 12 months	2.312	0.008**	1.005	0.476

*P < 0.05; **P < 0.01.

## Discussion

The notable differences in the clinical and radiologic features of patients with multiple brain metastases make the decision of optimal treatment much more difficult^3^. Most patients receive only WBRT treatment^14^. SRS can also be used to treat multiple brain metastases^15,16^. Surgical resection was previously considered to be unsuitable for patients with multiple brain metastases^8^. However, with improvements in surgical techniques, more aggressive surgical treatment has become more common for patients with brain metastases^17^. Several prospective and retrospective studies comparing surgery with SRS in the treatment of a single brain metastasis have not shown significant differences in either OS or local control rates between surgery and SRS^11,18,19^.

Only several retrospective reports addressing surgical treatment of multiple brain metastases have demonstrated a median OS of 10 to 14 months^7,20^. A retrospective report by Bindal et al.^21^ has found that the median OS of patients receiving total resection for multiple brain metastases and patients receiving total resection of single metastasis lesions was almost identical at 14 months, whereas the median survival of patients in whom only a portion of the multiple brain metastases was resected was significantly lower, at only 6 months (*P* < 0.05). Previous studies have also found that patients receiving surgical resection of multiple lesions show similar survival to patients undergoing surgical resection of a single metastasis. This finding remained true when neurological death was used for survival calculations^22,23^. Another study has revealed that surgical resection plus SRS for the surgical cavity in patients with a limited number of large brain metastases results in longer OS and better local control rates than SRS alone^24^.

No randomized clinical trials have compared surgical resection and SRS in patients with multiple brain metastases. Performing this type of prospective randomized trial is very difficult. Few patients are truly suitable for both surgery and SRS, owing to various factors, such as tumor size, location, the need for pathological diagnosis, the presence of elevated intracranial pressure, performance status, patient preference, or clinician bias. Given that patients present to different specialty clinicians, such a clinical trial would require close collaboration among neurosurgery, oncology, radiation oncology, and other medical units^5^.

Our findings suggest that, for patients with 2 to 4 brain metastases, surgery as the initial treatment is not inferior to SRS in terms of OS, although the tumor size in the surgery group was significantly larger than that in the SRS group.

The neurological death rate was very low in this study (2 patients in the surgery group and 6 patients in the SRS group). Overall, 89% of the patients died because of progression of the primary cancer, and OS as the primary endpoint was a relatively insensitive parameter. The local tumor control rate was excellent after both surgery (88.5%) and SRS (84.6%), and these rates did not significantly differ. This finding indicated the efficiency of local treatment of brain metastasis by either SRS or surgery. Advances in SRS, SRT, IMRT, and WBRT have continually increased the accuracy and conformity of radiotherapy^25^. The progress in surgical techniques and targeted therapy has also improved the outcomes of patients with brain metastasis^26–28^. The efficiency of anti-angiogenesis targeted therapy for radiation necrosis has also made physicians increasingly willing to treat patients aggressively with radiotherapy, such as SRS, IMRT, or WBRT^27,29,30^. Overall, this progress has made brain metastasis far less fatal.

Another major goal of brain metastasis treatment is to relieve related symptoms caused by the combination of the tumor mass and surrounding edema. Because the surgery group had much larger tumor sizes and more patients with symptoms, we performed propensity score matching to minimize the associated bias, and we generated 2 groups with similar proportions of patients with these symptoms. Neurologic symptoms were shown to consistently, but not significantly, improve more rapidly in the surgery group than the SRS group. A previous study has also shown that the resolution of edema is consistently and significantly faster with surgery than SRS^31^. However, the number of patients in our study was relatively small, thus potentially limiting the generalizability of our results.

Surgical resection of brain metastases has been reported to be associated with a higher incidence of leptomeningeal metastasis^32,33^. The risk of leptomeningeal metastasis in our cohort was comparable between the surgery group and the SRS group. A previous study has found that the risk of local recurrence is significantly higher with piecemeal resection than en bloc resection^34^. These results suggested that, when piecemeal resection is necessary, SRS should be the first choice management modality if piecemeal resection is inevitable, and postoperative WBRT or resection cavity SRS should be considered after piecemeal resection^35^.

Given the nature of prospective studies, the postoperative treatments in our group were decided case by case, on the basis of the risk of local failure and histologic sensitivity to radiotherapy or targeted therapy. The risk factors included pathologic diagnosis of small cell lung cancer, early signs of local failure on postoperative enhanced MRI, insufficient surgery margin, piecemeal resection, and the possibility of dissemination through the ventricular system or subarachnoid space in the surgery procedure. Patients with one of those risk factors usually receive WBRT or SRS or targeted therapy effective for brain metastasis. Patients with radio resistant tumors tend to receive surgery alone or surgery plus radiosurgery. The results from 2 randomized clinical trials have shown that surgery alone is insufficient to provide durable local control of brain metastasis^35,36^. Because approximately 50% of patients in the surgery alone group had local recurrence, some forms of radiotherapy are necessary after surgery. However, despite the finding that local control improved after SRS compared with observation, the OS was similar for both groups, thus suggesting that delayed radiotherapy, such as WBRT or SRS, still provides satisfactory local control after recurrence. Therefore, not all our patients received radiotherapy after surgery. The local control rate in our surgery group was 88.5%, which is comparable to the rates in other studies. Delayed radiotherapy may also decrease patients’ financial and physical burden and the risk of cognitive deterioration caused by radiotherapy.

## Conclusions

Our results suggest that surgical resection and SRS treatment for patients with multiple brain metastases have similar outcomes, such as OS and local control rate. Surgery appeared to provide faster symptom relief than SRS, but the difference was not statistically significant and requires a larger research cohort for confirmation. Various factors, such as age, progression status, status of the primary cancer, number of lesions, location, and size, were important during the treatment decision-making process for patients with multiple brain metastases. Therefore, the best treatment should be decided on a case by case basis.

As with all retrospective studies, even after propensity score matching, selection and exclusion biases could not be eliminated in this study. Therefore, we could not control for unmeasured or unknown variables, as could be achieved in a prospective randomized trial. Future studies with a larger number of patients are needed to confirm our findings.
